# Evidence for Cognitive Impairment in Mastocytosis: Prevalence, Features and Correlations to Depression

**DOI:** 10.1371/journal.pone.0039468

**Published:** 2012-06-20

**Authors:** Daniela Silva Moura, Serge Sultan, Sophie Georgin-Lavialle, Stéphane Barete, Olivier Lortholary, Raphael Gaillard, Olivier Hermine

**Affiliations:** 1 Centre de référence des mastocytoses, Hôpital Necker Enfants malades, Fondation Imagine Paris, Université Paris Descartes, Sorbonne, Paris Cité, Paris, France; 2 Université Paris Descartes, Sorbonne, Paris Cité, Laboratoire de Psychopathologie et Processus de Santé EA 4057, IUPDP Institut de Psychologie, Paris, France; 3 CNRS UMR 8147, Hôpital Necker Enfants malades, Paris, France; 4 Service de Médecine Interne, Hôpital Européen Georges Pompidou, Université Paris Descartes, Sorbonne, Paris Cité, Paris, France; 5 Département de dermatologie, Hôpital Tenon, Université Pierre et Marie Curie, Paris, France; 6 Université Paris Descartes, Sorbonne, Paris Cité, Service de maladies infectieuses et tropicales, Hôpital Necker Enfants malades, Paris, France; 7 Université de Montréal, Québec, Canada; 8 Centre de Recherche du CHU Sainte-Justine, Montréal, Québec, Canada; 9 INSERM; Université Paris Descartes, Sorbonne Paris Cité, Laboratoire de Physiopathologie des maladies Psychiatriques, Centre de Psychiatrie et Neurosciences U894, Paris, France; 10 Université Paris Descartes, Sorbonne Paris Cité, Faculté de Médecine Paris Descartes, Service Hospitalo Universitaire, Centre Hospitalier Sainte-Anne, Paris, France; 11 Université Paris Descartes, Sorbonne, Paris Cité, Service d’hématologie adulte, Hôpital Necker-Enfants malades, Paris, France; 12 Fondation Imagine, IHU Hôpital Necker-Enfants malades, Paris, France; Chiba University Center for Forensic Mental Health, Japan

## Abstract

Mastocytosis is a heterogeneous disease characterized by mast cells accumulation in one or more organs. We have reported that depression is frequent in mastocytosis, but although it was already described, little is known about the prevalence and features of cognitive impairment. Our objective was to describe the prevalence and features of cognitive impairment in a large cohort of patients with this rare disease (n = 57; mean age = 45) and to explore the relations between memory impairment and depression. Objective memory impairment was evaluated using the 3^rd^ edition of the Clinical Memory scale of Wechsler. Depression symptoms were evaluated using the Hamilton Depression Rating Scale. Age and education levels were controlled for all patients**.** Patients with mastocytosis presented high levels of cognitive impairment (memory and/or attention) (n = 22; 38.6%). Cognitive impairment was moderate in 59% of the cases, concerned immediate auditory (41%) and working memory (73%) and was not associated to depression (p≥0.717). In conclusion, **i**mmediate auditory memory and attention impairment in mastocytosis are frequent, even in young individuals, and are not consecutive to depression. In mastocytosis, cognitive complaints call for complex neuropsychological assessment. Mild-moderate cognitive impairment and depression constitute two specific but somewhat independent syndromes in mastocytosis. These results suggest differential effects of mast-cell activity in the brain, on systems involved in emotionality and in cognition.

## Introduction

Mastocytosis is a rare and heterogeneous disease characterised by accumulation of mast cells in one or several organs [1]–[3]. Based on organ dysfunction, systemic mastocytosis is divided into indolent (>90% of cases) and aggressive forms [3], [4]. Although in its indolent form mastocytosis is not a life threatening disease, deregulated mast cells activation and degranulation lead to the liberation of a panel of mediators (such as serotonin, histamine, tryptase, heparin, substance P, interleukins (IL8, IL4, IL10), TNF alpha…). Patients suffer from various clinical symptoms related to mast-cell degranulation and/or infiltration. These symptoms can be chronic (pruritus, urticaria pigmentosa, headache, articular and muscular pain, memory loss, attention impairment, depression) or paroxysmal (Flush, anaphylactic like episodes, syncope) [5]. Chronic symptoms of mastocytosis can be especially disabling and can significantly affect patients in their personal, social and professional life domains [6]. In this study, we report an assessment of chronic memory and attention impairment in mastocytosis and we explore the interrelationships with depression, age, education level, treatment and forms of the disease.

Cognitive complaints (memory and attention disturbances) are common in mastocytosis and in our recent work 38% of patients reported to feel concerned by these symptoms [6]. To date, only the study of Rogers and collaborators has evaluated cognitive impairments in 10 patients diagnosed with systemic mastocytosis by using a valid psychometric measure of memory function [7]. These authors brought for the first time evidence for memory and attention impairment in 70% of their sample of patients with systemic mastocytosis. They suggested that mast cells deregulation impact memory function through mediators released including histamine. Other neuropsychiatric symptoms such as depression are also present with high frequency in mastocytosis. The prevalence of depression was estimated 40%, 70% and 64% according to methods and cut offs used for investigation [6]–[8]. While prevalence and features of depression in mastocytosis has been recently well reported, cognitive (attention/memory) impairment still remains to be described.

Among neuropsychological symptoms coinciding with cognitive impairment, depression is the most frequent [9]. Although depression and cognitive impairment are common co-morbidities, the nature of this relationship and possible prognostic role of depression is still under debate [10]–[13]. In mastocytosis, cognitive impairment seems to be very frequent and since depression is a common symptom among our patients, it is critical to investigate both of these issues. In this work, we report an assessment of cognitive impairment (memory and attention) in a large sample of patients with mastocytosis (n = 57). In addition, we have investigated the relationship between depression, age, education, forms of the disease, treatment and cognitive impairment. We provide substantial evidence for high prevalence of cognitive impairment and we strongly suggest that further refinement of the assessment of memory and attention impairment in mastocytosis is needed.

## Results

### Subjective Complaints and Objective Cognitive Impairment are Frequent in Mastocytosis

In our sample (n = 57), 74% (n = 42) of patients reported a subjective complaint of cognitive impairment. Cognitive impairment (scores ≤85) concerned 38.6% of patients (n = 22). In this group, the mean (M) age was 42.31 (range = 20–66; standard deviation (SD)  =  12.08), 18 patients were under 53 years of age and only one was older than 65 years; 77% were women,. The education level was high (36% (n = 8) attended at least a first degree of graduation and 32% (n = 7) had a Master or PhD degree). Depression symptoms (Ham-D17≥12) were present in 68% (n = 15) of patients (M = 15.18, range = 0–35; SD = 9.09), 14 were taking an antihistaminic treatment, 1 patient was treated by imatinib, 5 were not taking any treatment, 3 were taking other symptomatic treatments (cromoglycates and/or biphosphanates) and 17 were taking psychotropic drugs including anxiolytic and antidepressants.

*Memory and attention impairments in mastocytosis are usually slight or moderate and are characterized by immediate auditory and working memory disabling.*

**Figure 1:**
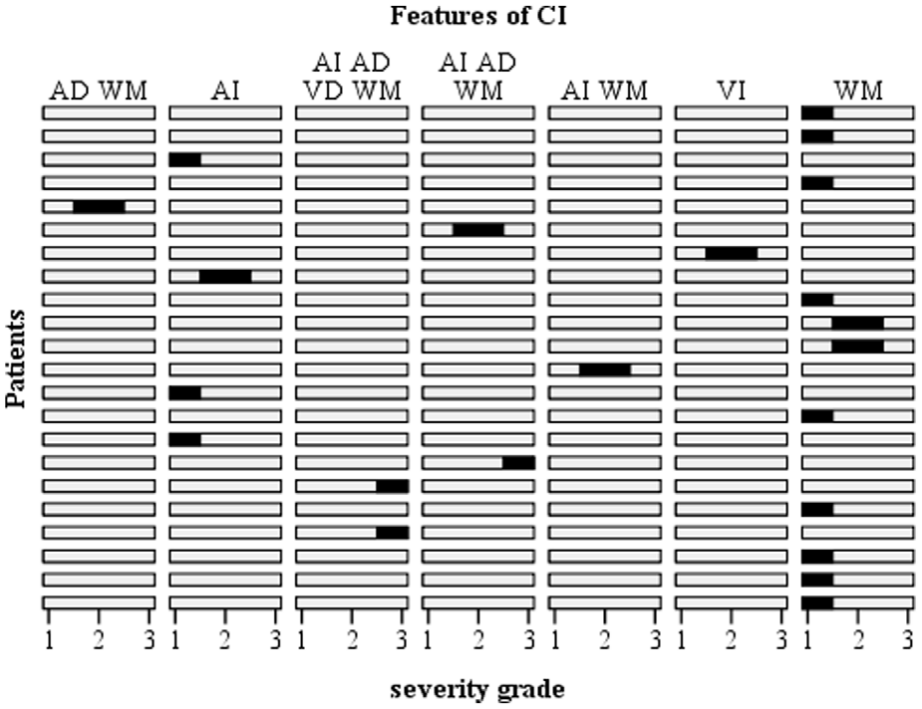
Features of cognitive impairment in mastocytosis. In the ordinate axis each line represents a patient. In the abscissa axis the severity of the cognitive impairment is represented for each column (1: slight; 2: moderate; 3: severe). Columns displays features of cognitive impairment observed (A: auditory; V: visual; I: immediate; D: delayed; WM: working memory). Black marks gives the feature and the severity of cognitive impairment for each patient. Among the 22 patients WM impairment as the most frequent concerning 16 patients (10 with WM alone and 6 associated to auditory and/or visual impairment. In our sample (n = 57), 22 patients (38.6%) were diagnosed with cognitive impairment. 12 patients (59.1%) have slight impairment; 7 (27.3%) have moderate impairment and severe impairment affected 3 (13.6%) patients.

Among our impaired patients (n = 22), the prevalence of slight (scores 85−80) and moderate (scores 79−71) cognitive impairment was 86.3% (n = 19). Severe impairment concerned only a minority of patients (n = 3). Figure 1 displays the features of cognitive impairment for each of the 22 diagnosed mastocytosis patients. Working memory (attention) impairment was the most frequent with 73% (n = 16) cases followed by auditory immediate memory impairment (41% (n = 9)). Auditory delayed memory and visual memory impairments concerned 31.8% (n = 7) of patients and appeared only in the context of auditory immediate and/or working memory disturbances (except for one patient). Patients presenting delayed memory impairment were significantly more impaired in attention (working memory) than patients without such impairment (p = 0.044) and presented more severe cognitive impairment (p = 0.012).

### Cognitive Impairment was Unrelated to Age, Education, forms of the Disease and Treatment in our Sample

Age did not distinguish our patients with respect to the presence of cognitive impairment (M = 42.31; S.D. = 12.08 vs M = 47.22; S.D. = 11.34; p = 0.134) but patients with severe impairment were significantly younger when compared to patients with slight or moderate impairment (M = 26; SD = 7.21 vs M = 45; SD = 10.64; p = 0.022). We found no significant correlation between the education level and the cognitive impairment (presence/severity (r = −0.179; p = 0.182/r = 0.181; p = 0.178, respectively). The mastocytosis clinical subcategories did not significantly correlate with cognitive impairment presence/severity (r = 0.218; p = 0.104/r = 0.227; p = 0.089 respectively). The percentage of patients with an antihistaminic treatment was not significantly different between patients with/without cognitive impairment (63.6% (14/22) vs. 74.3% (26/35)) and there was no significant correlation between the treatments used and the cognitive impairment (ps ≥0.148). Among impaired patients, 7 were taking psychotropic drugs and we found no correlation between these two issues (r = 0.035; p = 0.799). Similar results were obtained with subjective complaints (ps ≥0.070).

### Depression is not Related to the Presence or the Severity of Cognitive Impairment but Depressed Patients Presented Lower Scores in Delayed Visual Memory

**Figure 2:**
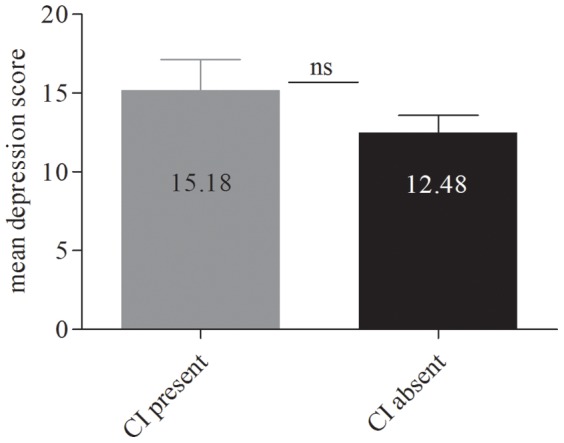
Cognitive impaired patients are no more depressed than patients without cognitive impairment. We performed an unpaired t-test to compare depression scores of patients with and without cognitive impairment. In the group of patients diagnosed with cognitive impairment mean depression score was 15.8 (SD = 9.09). In the group without cognitive impairment diagnosis the mean depression score was 12.48 (SD = 6.59).The analysis showed no significant difference between the groups (t = 1.296; p = 0.200). CI: cognitive impairment; ns: not significant.

Patients with/without cognitive impairment did not distinguish significantly neither in percentage of patients presenting depression symptoms (Ham-D17≥12) (68.2% (15/22) vs. 51.4% (18/35); p = 0.220) nor in depression level (M = 15.18; SD = 9.09 vs. M = 12.48; SD = 6.59; p = 0.236) (Figure 2). No significant correlation was found between depression (scores/presence) and the severity of cognitive impairment (r = 0.226; p = 0.092/r = 0.228; p = 0.088 respectively). In the impaired group (n = 22), depressed patients displayed slightly lower scores in delayed visual memory (M = 95.13; SD = 12.74 vs. M = 114.57; SD = 11.41; p = 0.003) but no significant difference was found for visual immediate memory (M = 112.14; SD = 7.73 vs. M = 99.44; SD = 15.7; p = 0.060), auditory memory (delayed: M = 96.71; SD = 8.99 vs. M = 91.46; SD = 14.06; p = 0.379; immediate: M = 93.28; SD = 10.27 vs. M = 88.44; SD = 16.87; p = 0.420) and working memory (M = 85.42; SD = 8.20 vs. M = 85.06; SD = 8.56; p = 0.926).

## Discussion

In this study, we show in a cohort of 57 patients that cognitive impairment is highly prevalent (38.6%) in mastocytosis. Disturbances in attention (working memory) and auditory immediate memory appeared as the most typical manifestations of cognitive impairment in these patients. These cognitive impairments were not related to depression, age, education level, or mastocytosis clinical subcategories. Furthermore, no correlation was found between cognitive impairment and antihistaminic intake, which is in line with data suggesting that although histamine seems to have a modulator effect in some mnemonic systems its exact function in memory is still controversial [14].

The prevalence of memory impairment in our sample was high but not as suggested by Rogers and collaborators (70% in a sample of 10 patients) [7]. Although we used the same instrument for diagnosing memory impairment, our study concerned a larger sample, which reduces the risk of selection bias and ensures more inter-variability. Our study provide evidence that the prevalence of memory impairment in mastocytosis is unrelated to age or education level (mean age of 42 years old and high education (32%) in the impaired group). In addition, the prevalence of cognitive impairment in our sample was significantly higher than in younger populations (45 to 59 years old) suffering from chronic somatic disease like diabetes or in elderly populations (≥65 years) in which prevalence of cognitive impairment-no dementia (CIND) is around 15%–40% [15]–[20]. Interestingly, the prevalence of this manifestation in our sample is similar than the one observed in multiple sclerosis, an inflammatory disease involving mast cells in which prevalence of cognitive impairment is estimated between 40% and 60% [21]–[23]. Furthermore, as reported above, although the rate of depression is high in our sample, we found no significant difference in the percentage of depressed patients with and without cognitive impairment (memory/attention (working memory) and no correlations between these two issues. Therefore, we could conclude that in mastocytosis patients cognitive impairment is not related to depression but rather to abnormal number and/or activation of mast cells. An hypothesis for the lack of correlation between cognitive symptoms and depression would be that specific mediators/cytokines released by deregulated mast cells (such as TNF-α or interleukin-6) would be required to provoke depressive symptoms while a broad range of mediators could impair cognitive process [24]–[26].

Only 7 patients were concerned with impairment in delayed memory. They appeared to be particularly impaired in working memory and 3 patients (significantly younger than the others) displayed severe cognitive impairment in this group. These results are in line with previous research in memory function suggesting that worse deficits in working memory can lead to severe disability as it can affect short and possibly long term memory domains [27]. Interestingly, the three patients displaying severe impairment where all young women (20, 24 and 34 years old). According to their medical records they had disorders of menstrual cycle including amenorrhea (without pregnancy, excessive exercise or anorexia), which was not a regular symptom among the other women patients in our sample and in mastocytosis in general. These three patients were also concerned with visual memory impairment, which affected exclusively women (5 patients concerned) in our sample. These observations could suggest that an involvement of mast cells in neuroendocrine regulation through acceleration of Gonadotropin-releasing hormone (GnRH) with influence in cognition could concern female patients with mastocytosis. This hypothesis is supported by several reports showing that brain mast cells store and secrete GnRH and that GnRH II, abundant in hippocampus and amygdales of primates and humans, has effects in cognition and particularly in visual memory performance in young women [28]–[32].

In the brain, mast cells are present in diencephalon, especially in hypothalamus, which is implicated in both emotion and cognition systems [33]–[35]. The implication of these immune cells in modulation of anxiety-like behaviour has been recently demonstrated in mice [36]. In human, several reports have suggested the implication of mast cells in anxious-depressive symptoms, in autistic spectrum disorders and in neurological diseases as Alzheimer and multiple sclerosis [8], [37]–[39]. Moreover, degranulation of brain mast cells has been linked to hypothalamic-pituitary-adrenal (HPA) responses via histamine release [40], [41]. Mast cells have also been shown to synthesise Corticotropin-Releasing Hormone (CRH) and to be activated by CRH [42], [43]. In addition, hyperactivation of HPA responses has been related to cognitive processes in pathological situations such as type 2 diabetes, multiple sclerosis and Alzheimer disease [44]–[46]. Altogether, these data suggest that mast cells are implicated in neuroimmunoendocrine responses. Therefore, alteration of the number or abnormal activation of these cells could potentially affect cognitive and emotional systems. In mastocytosis, several symptoms can be exacerbated by stress and a hyperactivation of HPA axis in patients presenting cognitive impairment is not excluded. Further research would be necessary to investigate this hypothesis.

There are few limitations to the current study. Even though the Wechsler Memory Scale is used in many studies and provides valid measures of memory functioning, a possible limitation to the results is the use of only one instrument to evaluate these impairments. Future research of memory impairment in mastocytosis should enlarge the panel of instruments used and include evaluation of executive functions. In addition, our study provided only cross-sectional data. A follow-up research will be necessary to investigate the causes of occurrence, and the evolution of these impairments. Nevertheless, this was the first description of memory and attention impairment in mastocytosis in a large sample of patients. We provided substantial evidence for high prevalence of these symptoms among these patients. Attention (working memory) and auditory immediate memory disturbances where identified as the most typical manifestations of cognitive impairment related to mastocytosis. Furthermore, we have shown that there was no significant relation between depression and these symptoms. Our results point to the need for further refinement of the assessment of memory and attention impairment in mastocytosis in a more systematic way. New pharmacological interventions through inhibition of mast cell degranulation by tyrosine kynase inhibitors (such as masitinib) could be used to treat cognitive impairment in these patients. This strategy has been shown to decrease mast cell degranulation related to symptoms in mastocytosis as well as cognitive impairment in Alzheimer disease. Another strategy would be the reduction of hippocampal neuroprotective kynurenic acid (KYNA) levels as proposed by recent studies in the treatment of Alzheimer disease [8], [47]–[50].

In conclusion, by evaluating cognitive functions in mastocyotsis our data provide evidences for the role of mast cells in brain functions. Targeting of mast cells could open new avenues in the treatment of neurological and psychiatric disorders.

## Methods

### Sample Description (Table 1)

Table 1 displays our patient's characteristics. Fifty seven consecutive patients (15 men/42 women) with mastocytosis diagnosis as defined by the WHO criteria [3] were evaluated between 2008 and 2011. Mastocytosis subcategories comprised indolent forms (cutaneous mastocytosis (n = 12), systemic mastocytosis (n = 41)) and aggressive mastocytosis (n = 4). Mean age on study was 45 years (range = 20–75); S.D. = 11.78) and only 3 patients were 65 years or older. Education level was high (Master or PhD degree) in 40% (n = 23). Most of patients (70%) were taking an antihistaminic treatment as symptomatic treatment and 58% (n = 33) were depressed (M = 13.52; range = 0–35; S.D. = 7.69).

**Table 1:** Sample description.

N = 57	N (%)	M, range (S.D.)
Age		45, 20–75 (11.78)
Women	42 (74)	
Men	15 (26)	
***Depression***		13.52, 0–35 (7.69)
Depressed(Ham-D17 score ≥12)	33(58)	
***Forms of mastocytosis***		
Indolent mastocytosis	53 (93)	
Aggressive mastocytosis	4 (7)	
***Treatments***		
Antihistaminic 1	24 (42)	
Antihistaminic 1 and 2	16 (28)	
Glyvec	1 (2)	
none	11 (19)	
Others (cromoglycates and/or biophosphanates)	5(9)	
Psychotropic (anxiolytic/antidepressants	17 (30)	
***Education***		
Primary (n = 1)/Secondary	13(23)	
Bachelor’s degree	21(37)	
Master or PhD degree	23(40)	

a Graduation level. M (mean); S.D. (standard deviation); N: number of patients.

The study was approved by Necker hospital ethical committee, and carried out according to the Helsinki convention. Each patient was informed about the study and provided written consent.

### Subjective and Objective Memory Impairment

Subjective memory complaints were measured by asking patients if they were concerned by memory difficulties (i.e. forgetfulness, difficulties in finding words, in remembering names, in concentrating and distractibility). Objective memory impairment was evaluated using the third edition of the Clinical Memory Scale of Wechsler [51]. This scale provides a standardised measure of important aspects of memory validated in French community. This instrument gives immediate and delayed scores for auditory and visual memory as well as scores for working memory that corresponds to attention capacities. Abnormal scores are considered from −1 standard deviation (SD) of the mean (M) T scores (100). Thus a score between 85 and 80 is reflecting a slight abnormality in memory function, whereas a score ≤70 (−2 SD) reflects a severe memory dysfunction. Objective cognitive impairment was diagnosed if the score was ≤85 in at least one of the memory dimensions evaluated as it is usually done in practice.

### Depression

Depression symptoms were measured with the 17 items Hamilton Depression Ranting Scale questionnaire (Ham-D17) [52]. Items were scored after a clinical interview focused on depression symptoms. The cut-off used to define presence of depression symptoms was Ham-D17≥12.

### Procedures

All patients were evaluated by the same licensed psychologist (DSM) in the French Reference Centre of Mastocytosis in Paris. An interval of 20 minutes was made between the end of part 1 (immediate recall) and the start of part 2 (delayed recall) of the memory test and patients were than screened for depression symptoms and asked about their memory complaints. Working memory was evaluated at the end of the part 1.

### Statistical Analysis

SPSS version 17.0 (IBM SPSS Inc., Chicago, IL, USA) was used for descriptive and correlation analysis in relation to forms of mastocytosis and education level. Mann Whitney test for nonparametric data and unpaired t test used to compare groups in relation to cognitive impairment, depression, age, and treatment were performed using GraphPad Prism software version 5.01 (GraphPad Software Inc., San Diego, CA).
